# Rosacea Granulomatosis in a Neutropenic Leukemic Patient

**DOI:** 10.7759/cureus.23308

**Published:** 2022-03-19

**Authors:** Woo Joo Lee, Natan Kraitman, Carlos J Sarriera-Lazaro, John Greene

**Affiliations:** 1 Infectious Disease, H. Lee Moffitt Cancer Center, Tampa, USA; 2 Infectious Disease, Sarasota Memorial Hospital, Sarasota, USA; 3 Dermatopathology, College of Medicine, University of South Florida Morsani, Tampa, USA

**Keywords:** acute myeloid leukemia, neutropenia, immunocompromised, demodex folliculorum, rosacea granulomatosis

## Abstract

Rosacea granulomatosis is a common, chronic skin disorder that primarily affects the central face, namely the cheek, nose, chin, and central forehead. Although rosacea is mainly a disorder of innate and adaptive immunity, a variety of endogenous and exogenous triggers such as *Demodex* may stimulate it. Often found as commensal organisms in human skin, *Demodex* ​​​​​​​can be parasitic if there is a change in the host’s cutaneous environment. This is especially relevant for immunosuppressed patients, who need prompt treatment to prevent further complications. We review the literature regarding rosacea granulomatosis in immunosuppression and present an acute myelogenous leukemia patient with severe neutropenia, which may have promoted the development of rosacea due to *Demodex* ​​​​​​​mite proliferation. This local proliferation of the ectoparasite on the face can cause an atypical skin rash that mimics severe infections in the setting of neutropenia.

## Introduction

Rosacea granulomatosis is a chronic skin disease that mostly involves the cheeks, nose, chin, or forehead [1]. The exact cause of rosacea granulomatosis is unrevealed, but several trigger both genetics and nongenetic are known and infectious organisms including *Demodex *and some bacteria are possible causal factors [2]. Clinical manifestations of the disease could be hard to differentiate from other dermatologic disorders. Therefore, the differentiation of rosacea granulomatosis from other skin infections that mimic rosacea is crucial. We present a patient with acute myeloid leukemia, who developed rosacea granulomatosis with severe neutropenia after chemotherapy.

## Case presentation

**Figure 1:**
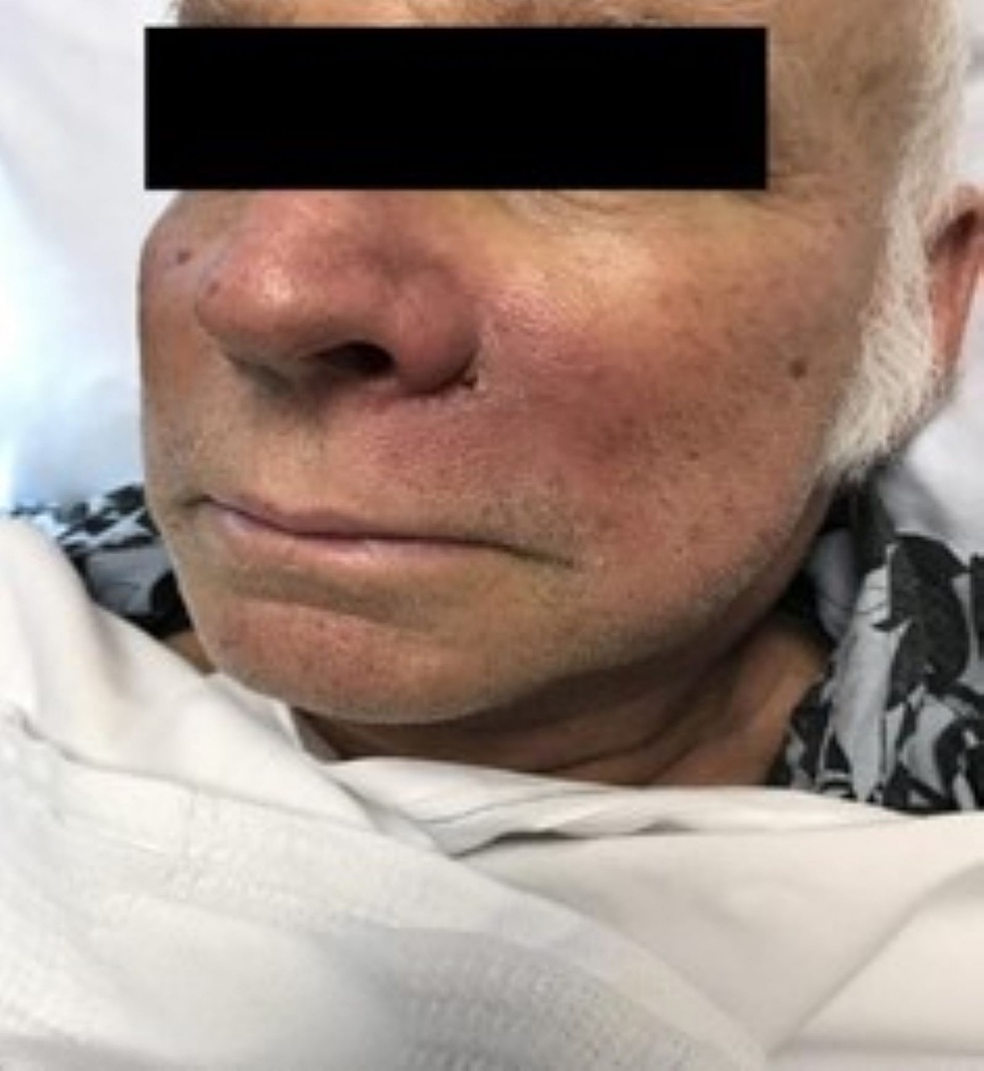
A plaque close to the left nasal sidewall with yellow crusting with surrounding erythema

A 69-year-old male patient with acute myeloblastic leukemia, who was undergoing induction chemotherapy, developed neutropenia. The initial absolute neutrophil count ranged from 650 to 1350 cells/mm^3^. The patient ultimately developed severe neutropenia, with the neutrophil count dropping below 500 cells/mm^3^. Therefore, he started on routine prophylaxis with oral acyclovir, ciprofloxacin, and voriconazole. On day 6 of severe neutropenia, the patient developed erythema extending from the left nasal sidewall, nasolabial fold, and left cheek, with induration. A plaque close to the left nasal sidewall with yellow crusting was also noted (Figure 1).

**Figure 2:**
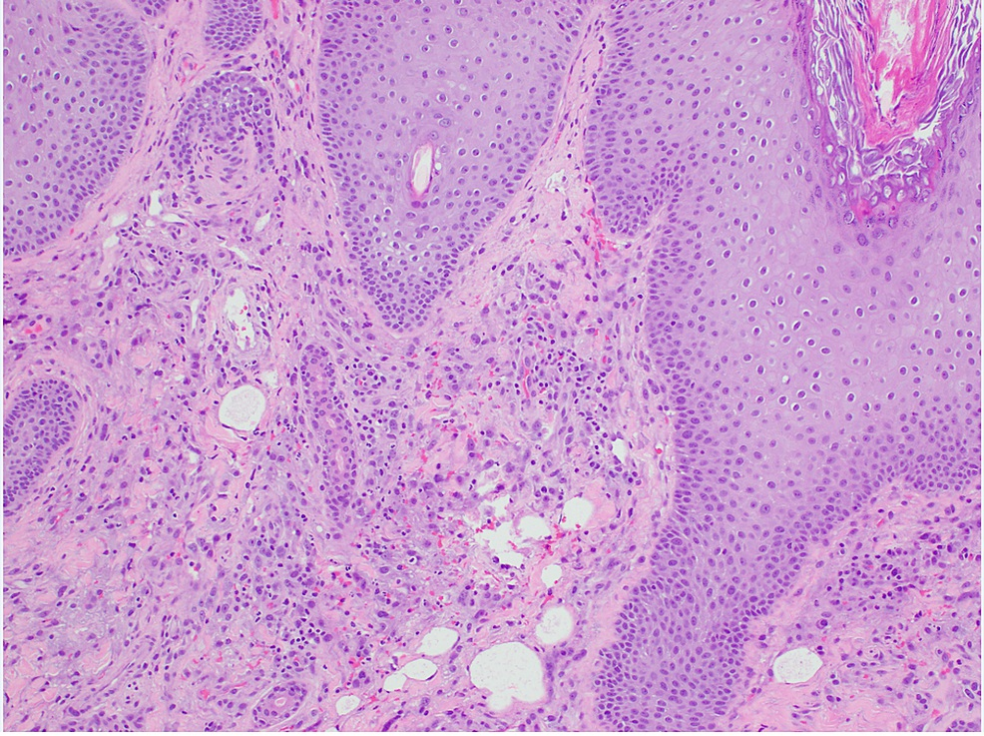
Granulomatous dermatitis with perifollicular and sparsely diffuse inflammatory infiltrate composed of histiocytes and lymphocytes

Several dermatologic diseases associated with microbial infection such as scabies, mold skin infection, and ecthyma gangrenosum were suspected. Due to the progressive induration while on prophylaxis, therapy was escalated to vancomycin, cefepime, Flagyl and liposomal amphotericin B. CT scan of the sinuses revealed right maxillary sinus inspissated mucus and left soft-tissue thickening. A biopsy of the area revealed granulomatous dermatitis most consistent with granulomatous rosacea. The histological changes were mild and nonspecific. A mixture of histiocytes and plasma cells was present in the infiltrate (Figure 2).

All tissue cultures remained negative. Given that granulomatous rosacea with the histologic finding usually linked to an increased number of *Demodex *mites, the patient was treated with an initial dose of ivermectin at 200 mcg/kg followed by a repeat dose after 7 days. The oral route ivermectin was chosen instead of the topical route due to the patient’s immunocompromised condition. This resulted in marked improvement and resolution of the erythema prior to the resolution of neutropenia.

## Discussion

Rosacea is a common, chronic skin disorder that affects approximately 18% of the population worldwide [3], with an increased incidence in people of Celtic origin [4]. Although the history of rosacea is not clearly known, it is said to have been mentioned by Theocritos in the third-century BC. A medical term “gutta rosacea” was documented by J. Plenck (1735-1807), who separated this term from “vari” or “ionthi” in the classification of skin diseases [5]. Rosacea primarily affects the central face, namely the cheek, nose, chin, and central forehead. According to a report developed by the National Rosacea Society Expert Committee on the Classification and Staging of Rosacea, there are primary and secondary features of rosacea. This group recognizes four patterns of signs and symptoms, which are designated as the following subtypes. Subtype 1 erythematotelangiectatic rosacea is characterized by persistent, central facial erythema, flushing, and telangiectases. Such characteristics, although common in this subtype, are not essential for a diagnosis. Subtype 2 papulopustular rosacea includes persistent, central facial erythema. This subtype may present with transient papules, pustules, or both. Patients have also reported associated burning and stinging. Subtype 3 phymatous rosacea can be associated with thickening of the skin, irregular surface nodularity, and enlargement. This subtype most commonly presents as an enlarged, bulbous nose, or rhinophyma. Subtype 4 presents as ocular rosacea [6]. Ocular manifestations may include conjunctivitis, blepharitis, and inflammation of the eyelids and meibomian glands.

Although rosacea is mainly a disorder of innate and adaptive immunity, it can be stimulated by a variety of endogenous and exogenous triggers [7,8]. With regards to subtype 2, papulopustular rosacea, most experts have distinguished two causes: papulopustular rosacea, not caused by *Demodex*; rosacea-like demodicosis, caused by *Demodex* [7,8,9]. *Demodex folliculorum* (*D. folliculorum*) and *Demodex brevis* are two species that can be found in humans [10]. *Demodex *mites are generally considered commensal organisms in human skin; however, they can be parasitic if there is a change in the host’s cutaneous environment, which enables their proliferation [11,12]. A retrospective analysis revealed that there is a significant association between *Demodex *infestation and the development of rosacea [13]. Ivy et al. reported on opportunistic infection of the skin by *Demodex *in immunocompromised children undergoing chemotherapy for leukemia. The authors attributed this to an increase in mite proliferation, as a result of depletion of cell-mediated immunity secondary to lymphocyte depletion [14]. A retrospective study reported the incidence of papulopustular lesions on the cheeks of patients following chemotherapy with epidermal growth factor receptor inhibitors. These patients were found to have an increase in *D. folliculorum* in the cheek. This, according to the authors, was attributed to a reduction or impairment of the cutaneous defense mechanisms by the epidermal growth factor receptor inhibitors, resulting in an increase in *Demodex *proliferation [15].

**Figure 3:**
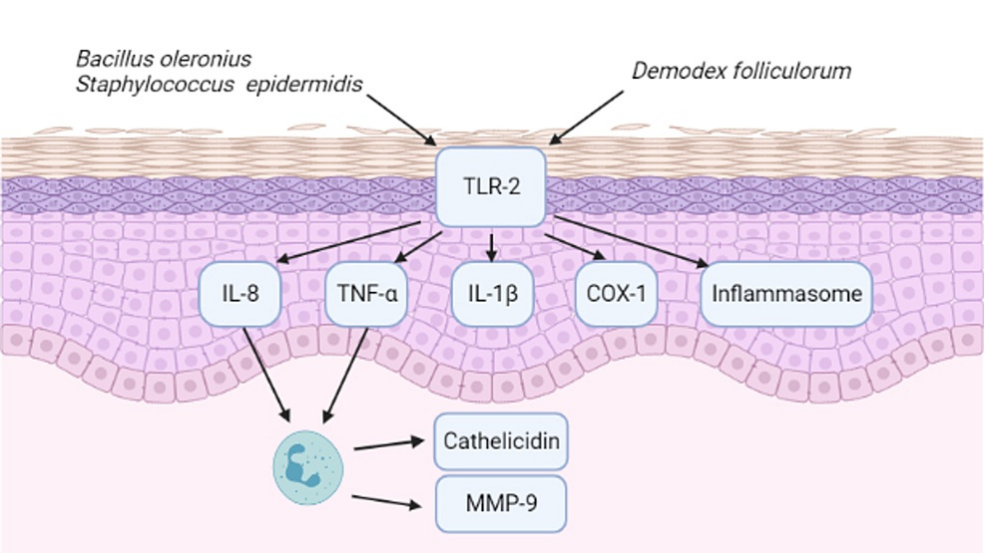
Pathophysiology of rosacea caused by microbial stimulation *Demodex folliculorum*, *Bacillus oleronius,* and *Staphylococcus epidermidis* prompt inflammatory responses from keratinocytes through the toll-like receptor-2 (TLR-2) pathway [24]. This enhances the expression of inflammatory mediators such as IL-8, TNF-α, IL-1β, COX-1, and other inflammasomes. These cytokines aggravate inflammatory reactions and are also involved in angiogenesis. In addition, neutrophils induced by IL-8 and TNF-α could release MMP-9 and cathelicidin, which worsen the inflammation. TLR-2: toll-like receptor-2; MMP-9: matrix metalloproteinase-9; COX-1: cyclooxygenase-1; TNF-α: tumor necrosis factor-α

It has been proposed that *Demodex *mites may cause skin conditions by several pathogenic mechanisms: *Demodex *mites are known to parasitize the pilosebaceous unit, they may block the pilosebaceous unit resulting in epithelial hyperplasia and hyperkeratinization [16]; the glandular and epithelial cells lining the hair follicles may be damaged by the enzymatic activity of the *Demodex *mite, resulting in inflammation [17,18]; *Demodex *mites contain lipase, which results in the conversion of sebum into components that are cytotoxic and irritant to the skin [19]; thus triggering an immune reaction due to the presence of parasitic antigens [20]. Toll-like receptor-2 (TLR-2), a kind of pattern recognition receptor, is upregulated in rosacea patients, and *Demodex *mites are thought to be the trigger of the receptor’s activation and stimulate inflammation in rosacea (summarized in Figure 3) [21]. Microbiota, *Bacillus oleronius*, residing on *Demodex *mites and *Staphylococcus epidermidis* antigens could be a trigger as well [22,23]. The pro-inflammatory response of keratinocytes stimulated by TLR-2 produces a proliferation of mononuclear cells and interleukin (IL)-8, tumor necrosis factor-α (TNF-α), interleukin (IL)-1β, and other inflammasomes [21]. The pathophysiology of rosacea stimulated by microbes is shown below (Figure 3).

Ivermectin (22,23-dihydroavermectin B1) is a broad-spectrum antiparasitic drug that belongs to the family of macrocyclic lactones known as avermectins. Avermectins are produced by cultures of the filamentous bacterium *Streptomyces avermitilis* [25]. As an antiparasitic agent, ivermectin acts to increase the cell permeability to chloride ions, which in turn polarizes the nerve and muscle cells, thus paralyzing and killing the organism. Ivermectin acts on parasites, by showing the crystal structure of the glutamate-gated chloride channel receptor binding to ivermectin [26]. Several studies have shown the potent activity of ivermectin against numerous itch mites [27]. Brown et al. reported a case of papulopustular facial lesions, accompanied by ocular changes, that was refractory to corticosteroid and cyclosporine therapy. After a single 12-mg dose of ivermectin at 250 μg/kg, the patient’s symptoms resolved, with no reported recurrence [28]. Other rashes that can mimic rosacea include seborrheic dermatitis, acne, atopic dermatitis, erysipelas, systemic lupus erythematosus, and slapped cheek due to parvovirus B19. And some rosacea mimicking rashes are also associated with microbiota and require antimicrobial treatment and other agents to manage them.

In neutropenic patients, rashes that mimic rosacea that can be life-threatening include ecthyma gangrenosum usually due to *Pseudomonas aeruginosa*, mold infection, and nontuberculous mycobacterium skin infection. Noninfectious rashes in leukemic patients that can mimic infection include leukemia cutis, Sweet’s syndrome, and neutrophilic eccrine hidradenitis [29]. In terms of prevention for patients who experienced rosacea granulomatosis caused by *Demodex*, cleansing the face twice daily with non-soap cleanser, avoiding oil-based cleansers and greasy makeup, and exfoliating periodically to remove dead skin cells could be effective [30].

## Conclusions

We report a case of acute myelogenous leukemia patient with severe neutropenia, of which his immunosuppressed condition may have promoted the development of rosacea due to *Demodex* mite proliferation. The treatment commonly used in rosacea did not relieve his symptom until the use of ivermectin. From this case, we could discuss several rashes caused by infectious and noninfectious origins that mimic rosacea. There can be a variety of triggers that can stimulate rosacea granulomatosis; hence, we suggest immunosuppressed patients presented with rash to be considered infectious organisms as triggers and to be managed promptly.
